# Radiographical Evaluation of Morphological Alterations of Mandibular Third Molars: A Cone Beam Computed Tomography (CBCT) Study

**DOI:** 10.7759/cureus.34114

**Published:** 2023-01-23

**Authors:** Harsh Priyank, Butta Viswanath, Ankur Sriwastwa, Prashant Hegde, Nishath Sayed Abdul, Mahesh Suganna Golgeri, Shivakumar G C, Hemant Mathur

**Affiliations:** 1 Department of Conservative, Endodontics & Aesthetic Dentistry, Dental College, Rajendra Institute of Medical Sciences, Ranchi, IND; 2 Endodontics, Dental Care Centre, Ranchi, IND; 3 Department of Conservative Dentistry and Endodontics, Awadh Dental College and Hospital, Jamshedpur, IND; 4 Department of Oral and Maxillofacial Surgery, Century Dental College, Kasargod, IND; 5 Department of Oral and Maxillofacial Surgery and Diagnostic Sciences, College of Dentistry, Riyadh Elm University, Riyadh, SAU; 6 Department of Prosthodontics, Riyadh Elm University, Riyadh, SAU; 7 Department of Oral Medicine and Radiology, People’s College of Dental Science and Research Centre, Bhopal, IND; 8 Department of Oral Medicine and Radiology, Pacific Dental College and Hospital, Udaipur, IND

**Keywords:** endodontics, molars, topography, morphology, roots

## Abstract

Introduction

Morphological changes or variations in the lower third molar can be of concern during the endodontic, orthodontic, or prosthetic intervention. The goal of the present study was to evaluate the morphological alterations in the roots and root canal of a mandibular third molar in Bhopal, Central India, on cone beam computed tomography (CBCT) images.

Methodology

CBCT scans of 277 mandibular molars, of both genders, between the ages of 18 and 60 years were assessed for the presence of root numbers, the configuration of the canal based on Vertucci’s categorization, and a C-shaped canal. Scan results were analyzed for differences in canal configuration between the roots and topographical distribution. A chi-square test was applied to find any significant differences between the teeth at p 0.05.

Results

Scans analyzed for variations in the third molar had a mean age of 38.64 + 5.71 years. The majority (95.3%) of the molars had two roots, 1.5% had three roots, and 0.4% had five roots. The mesial side of double-rooted teeth predominantly had Type II canal configuration (67.0%), while it was Type I (79.2%) in the distal aspect of the root. C-shaped canals were detected in 21 teeth, and no significant topographical difference was noted in the CBCT images.

Conclusion

The majority of the current population showed two roots with the same number of canals in the studied tooth. CBCT can be used as a diagnostic aid in identifying the canal numbers and their configuration so as to render appropriate intervention and minimize subsequent failure.

## Introduction

The anatomical study of roots and canals has both clinical and anthropological significance. A thorough understanding of the root canal shape is essential for successful root canal therapy. Identifying the variances in tooth structure and characteristics across different racial groups is essential because it can help in the location, re-negotiation, and management of canals at the time of pulpal therapy [1]. The root canal system has been categorized under several headings owing to its intricacy. Vertucci's classification is one such description that is most commonly used by researchers [2].

Molars are particularly crucial as they are a frequent location for pulpal and periapical diseases. Ethnic differences, origin, age, and gender appear to be some genetic variables that influence the morphology of molars [3]. Nevertheless, the methodological process and identification method of the study can also influence anatomical variability. The configurations of root canal systems can be examined through both in vitro [4,5], ex vivo [6], and in vivo [7] techniques. Cone-beam computed tomography (CBCT) imaging is a recent development, and it offers a non-destructive, three-dimensional (3D) method for obtaining accurate data on the tooth structure. It allows for reduced radiation and the benefit of real-time performance throughout the clinical endodontic treatment. In addition, when compared to other radiographic techniques, CBCT imaging is more sensitive, detects anatomic changes more frequently, and offers significantly more details [8].

Third molars are integral to the specialties of endodontics, prosthodontics, and oral surgery. In endodontics, they mainly aim to preserve the functional part of the arch. Third molar autotransplantation can be tried to replace teeth if they cannot be restored. Mandibular third molar tooth autotransplantation is said to be a successful surgical procedure to replace non-restorable teeth with a high long-term survival rate [9]. In prosthodontics, they are retained for use as abutment teeth in fixed partial dentures. Thus, the morphological observation of the mandibular third molar is crucial for various procedures. An in-depth understanding of the canal morphology will help the dentist avoid procedural errors and treatment failures, especially in the thinnest area.

Literature reporting mandibular third molar morphology using CBCT scanning is scarce in this part of the country. Hence the current study was undertaken to answer the research questions, "What is the variation in the number of mandibular third molar roots in the study population?" and "What is the morphological variation in the canal of the mandibular third molar among the study population?"

## Materials and methods

A retrospective study was conducted to assess morphological variations of lower third molars in the roots among CBCT scans taken at Pratibimb Diagnostic imaging center, Bhopal, for various dental purposes from November 2021 to June 2022.

Sample selection and CBCT imaging

The study included an assessment of CBCT scans from 264 patients, in the age range of 18 to 60 years, of both genders. The sample size was calculated by the population and the patient reporting to the department. CBCT scans were performed as part of these patients' routine evaluation, diagnosis, and treatment planning. Informed consent of every individual was obtained telephonically, and the confidentiality of every participant was ensured. A convenient sampling technique was chosen for the present study.

Permission to carry out the study was obtained from the Institutional Ethical Committee of People's College of Dental Sciences and Research Centre, Bhopal (EC/2021/03)

Third molars on the right and left sides of the mandible were assessed in every image. Mandibular third molars that were fully erupted and had roots that were fully developed without evidence of periapical lesions, endodontic therapy, or a patent apex were included. Teeth in any position were included either be it buccoverted or lingoverted. Retained deciduous molars, restorations, posts, or crowns, teeth with calcified roots or root resorption, and molars that had undergone endodontic treatment were excluded.

Images were scanned using the 9300 3D CBCT equipment (Carestream Health, New York, USA). Parameters for the images included: a field of view of 10 cm, 90 kV (kilovoltage), 4 mA (Milliamperes), a voxel size of 0.18 mm, a scan time of 8 seconds, and data that were restructured at an interval of 1 mm-wide slice. Four experts evaluated the images in a room with low lighting. No more than three consecutive scans were carried out by a single examiner without a pause in order to avoid visual fatigue. With the use of Carestream software, the scans were examined and reviewed on a CS 3D Imaging software version 6.1.4 (Carestream Health, New York, USA). Evaluators even had the option to enlarge photos and alter viewing parameters like density, contrast, and sharpness.

Calibration and reliability of examinations

Three experts from the department of oral medicine and radiology looked for details in the CBCT scan. In case of any disagreement, it was sorted out by an oral radiologist. The interobserver agreement values (kappa for canal system configuration and intraclass correlation coefficient for the number of roots and number of canals) obtained by the two experts after they individually graded 264 CBCT pictures were all greater than 0.82, which is regarded as an adequate value. Test-retest reliability was also performed on 20 scans, and the scores all ranged above 0.77, ensuring adequate consistency.

Outcomes evaluated 

Each scan was evaluated for the number of roots and canals per tooth and the frequency of C-shaped canals in the third molar on the mandible.

Data analysis 

IBM SPSS Statistics for Windows, Version 23 (Released 2015; IBM Corp., Armonk, New York, United States) was used to statistically analyze the collected data. To determine the statistical significance of the comparisons, certain statistical tests were used to compare the data. To confirm the data's normality, the Kolmogorov-Smirnov test was applied. Numbers and percentages were used to compare the variables. Vertucci categorization differences between the number of roots were determined using the chi-square test, with a level of significance set below 0.05.

## Results

This imaging study was conducted to assess the variations and configuration of the mandibular third molar on the CBCT scans. The mean age of the study population was 38.64 + 5.71 years. Here, 56.3% of the scans belonged to the male population while 43.7% to females. The majority of scans were recommended for the orthodontic purpose (35.1 %), followed by periodontal reasons in 30.3%, implants in 20.5%, and other reasons in 14.1% of the cases as seen in Table 1.

**Table 1:** Characteristics of scans included

Characteristics	Mean + standard deviation
Age	38.64 + 5.71
Gender	N (%)
Males	156 (56.3 %)
Females	121 (43.7 %)
Reasons for scans	N (%)
Orthodontic	97 (35.1 %)
Periodontal related	84(30.3 %)
Implants	57(20.5 %)
Others	39(14.1 %)

**Figure 1:**
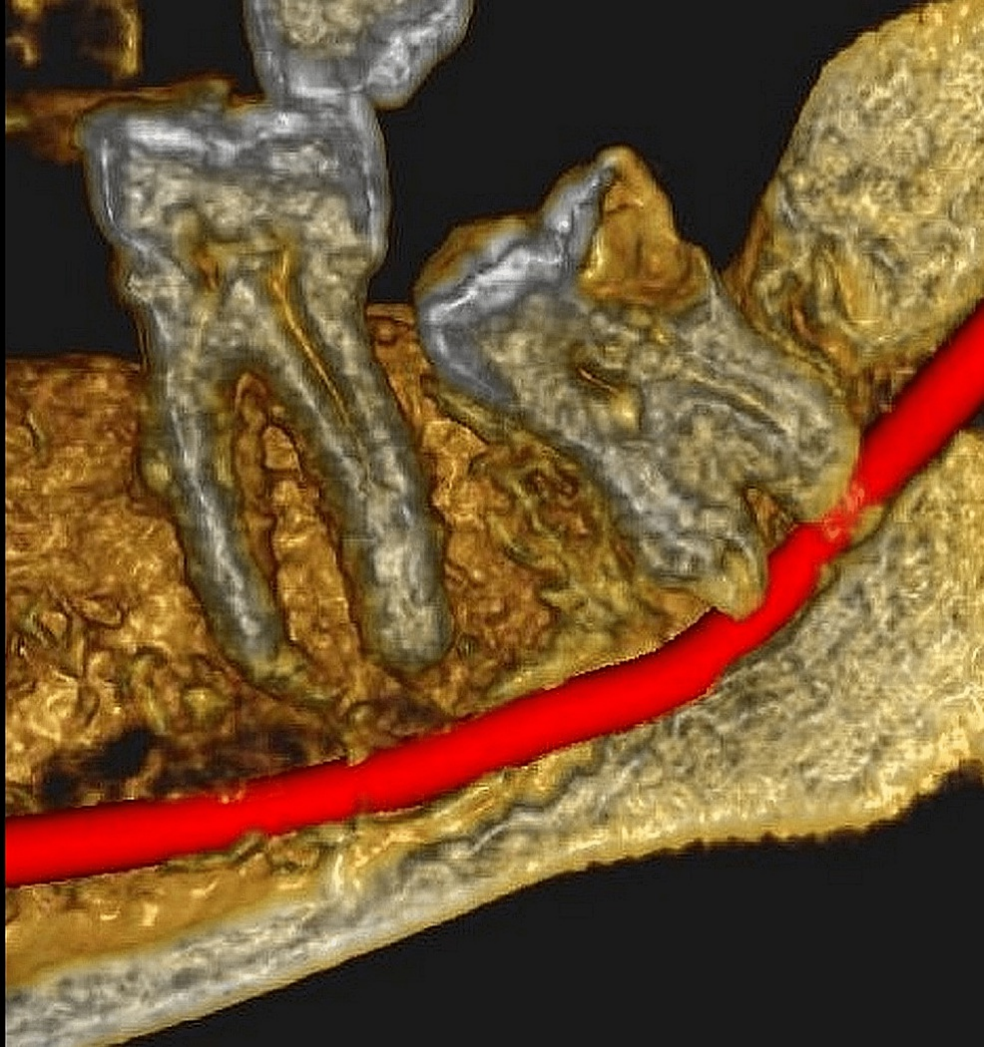
Picture showing canals in mandibular molar as a three-dimensional construction of a cone beam computed tomography

Of the total 277 scans assessed, 8 (2.8%) of them had single roots, 264 (95.3 %) presented with two roots, four (1.5 %) scans with three roots, and one (0.4%) with five roots (Figure 1).

The most commonly found canal category in single-rooted teeth is Type I followed by Type II and Type III. Among the two root categories, the mesial root presented predominantly with Type II canal in 67.0% of scans while the distal root had an inclination to Type I canal as reported in 79.2%, which was statistically significant at p=0.000. The second most commonly prevalent canal type was Type III (16.3%) in the mesial root and Type II (11.0%) in the distal root of the two rooted mandibular teeth. When assessed for three roots, the mesial root presented with Type II in 75.0% of images and Type III in 25%; all of the distal roots presented only with type I, and a greater proportion of distolingual root with Type I (75.0%), which was significant at p=0.030. On evaluating the tooth with five roots, the buccal side showed a Type III canal, mesiobuccal, and distobuccal with Type IV, while both lingual roots had a Type III pattern. This suggests wide variation in the type of canals between the roots (Table 2).

**Table 2:** Vertucci distribution among different rooted mandibular third molars *: statistically significant NS: Not significant

Number of Roots	Side	Type I	Type II	Type III	Type IV	Type V	Type VI	Total	Chi – square statistic	P value
1	-	5 (62.5)	2 (25.0)	1(12.5)	0 (0.0)	0 (0.0)	0 (0.0)	8		
2	Mesial	23 ( 8.7)	177 (67.0)	43 (16.3)	14 ( 5.3)	5 (1.9)	2 (0.8)	264 (100.0)	279.420	0.000*
	Distal	209 ( 79.2)	29 ( 11.0)	10 (3.8)	8 ( 3.0)	3 ( 1.1)	5 (1.9)	264 (100.0)		
3	Mesial	0 (0.0)	3 (75.0)	1 (25.0)	0 (0.0)	0 (0.0)	0 (0.0)	4 (100.0)	10.714	0.030*
	Distal	4 (100.0)	0 (0.0)	0 (0.0)	0 (0.0)	0 (0.0)	0 (0.0)	4 (100.0)		
	Distolingual	3 (75.0)	0 (0.0)	1 (25.0)	0 (0.0)	0 (0.0)	0 (0.0)	4 (100.0)		
5	Buccal	0 (0.0)	0 (0.0)	1 (100.0)	0 (0.0)	0 (0.0)	0 (0.0)	1 (100.0)	5.000	0.287(NS)
	Mesiobuccal	0 (0.0)	0 (0.0)	0 (0.0)	1 (100.0)	0 (0.0)	0 (0.0)	1 (100.0)		
	Distobuccal	0 (0.0)	0 (0.0)	0 (0.0)	1 (100.0)	0 (0.0)	0 (0.0)	1 (100.0)		
	Mesiolingual	0 (0.0)	0 (0.0)	1 (100.0)	0 (0.0)	0 (0.0)	0 (0.0)	1 (100.0)		
	Distolingual	0 (0.0)	0 (0.0)	1 (100.0)	0 (0.0)	0 (0.0)	0 (0.0)	1 (100.0)		

When compared for the bilateral symmetry among double and three-rooted teeth, no significant differences were found between the right and left sides in all the scans as seen in Table 3, suggesting that topographical variation was not appreciated among the scans. Both the right and left sides had a similar type of canal categorization.

**Table 3:** Vertucci distribution of mandibular third molar based on topography NS: Not Significant

Number of Roots	Root	Topographical side	Type I	Type II	Type III	Type IV	Type V	Type VI	Total	Chi – square statistic	P value
2	Mesial	Right	12 (7.9)	107 (70.4)	23(15.1)	6 (3.9)	3 (2.0)	1 (0.7)	152	0.937	0.967 (NS)
		Left	11 (7.2)	114 (75.0)	19 (12.5)	5 (3.3)	2(1.3)	1 (0.7)	152		
	Distal	Right	124 (81.6)	13 ( 8.6)	8 (5.3)	4 (2.6)	1 (0.7)	2 (1.3)	152	1.189	0.946 (NS)
		Left	119 (78.3)	15 (9.9)	12 (7.9)	3 (2.0)	1 (0.7)	2 (1.3)	152		
3	Mesial	Right	0 (0.0)	1 (50.0)	1 (50.0)	0 (0.0)	0 (0.0)	0 (0.0)	2	0.000	1.000 (NS)
		Left	0 (0.0)	1 (50.0)	1 (50.0)	0 (0.0)	0 (0.0)	0 (0.0)	2		
	Distal	Right	2 (100.0)	0 (0.0)	0 (0.0)	0 (0.0)	0 (0.0)	0 (0.0)	2	Not computed	-
		Left	2 (100.0)	0 (0.0)	0 (0.0)	0 (0.0)	0 (0.0)	0 (0.0)	2		
	Distolingual	Right	1 (50.0)	0 (0.0)	1 (50.0)	0 (0.0)	0 (0.0)	0 (0.0)	2	1.333	0.248 (NS)
		Left	2 (100.0)	0 (0.0)	0 (0.0)	0 (0.0)	0 (0.0)	0 (0.0)	2		

A total of 21 scans were reported with C canal configuration, with 80.5% with Continuous C shape canal followed by semicolon shaped canal in 9.5% as seen in Table 4.

**Table 4:** Distribution of C canal based on their configuration among scans

C canal configuration	N	%
Continuous C shape canal	17	80.5
Semicolon shape canal	2	9.5
Separated canal	1	5.0
One round or oval shaped canal	1	5.0
Total	21	100.0

## Discussion

A thorough understanding of the morphological aspect of the root canal system is vital for endodontic therapies. Endodontic failures are generally attributed to missing canals and improper instrumentation. Lower third molars exhibit greater heterogeneity in root patterns and canal shape [1]. Ethnicity and genes significantly influence morphological variations in root canals [10]. Hence, the need arises for research in various racial groups to assess differences in tooth morphology. The Negritos, Proto-Australoids, Mongoloids, and Mediterraneans are the four racial groupings that currently make up the Indian subcontinent's population. The Proto-Australoid race perhaps arrived shortly after the Negritos. Aboriginal Australians serve as their sources. They have established themselves in various locations in Central India [11]. 

Various approaches were evaluated for the study of the root canal system such as the use of CBCT, peripheral quantitative CT, spiral CT, plain (plain digital), contrast medium-enhanced digital radiographs, canal staining, and tooth cleaning techniques [12]. Literature evidence suggests the tooth-clearing technique and canal staining as the gold standard procedure. CBCT is found to be equally accurate and effective both in vitro and in vivo and also renders the benefit of being a noninvasive method [13]. CBCT is the suitable approach as the three-dimensional evaluation of root canal morphology can identify the majority of distolingual roots and C-shaped canals.

On a conventional radiograph, it is difficult to visualize the third roots of mandibular molars because the roots themselves are thin and the pictures of the roots overlap. It requires multiple exposures in conventional radiography, but CBCT is without any magnification and can precisely check the root positioning. Additionally, it may become difficult to see where they branch if the canals are fine. It may be beneficial to obtain an angled view (both vertically and horizontally), although obtaining one is difficult [14]. There is considerably greater overlap if there are nearby teeth, restorations, or implants, which lessens the sharpness of the radiography image. If root canal therapy is indicated, the presence of an extra distolingual root needs special consideration, particularly if they are smaller and more curved than the other roots. Absence to locate the confined extra distolingual channel and subsequent failure of navigation results in the failure of root canal therapy.

The present study showed that 2.8% of the scans had single roots, 95.3 % had two roots, 1.5 % of scans presented with three roots, and 0.4% showed five roots. The study of Park et al showed similar results with respect to the presence of three roots in 1.9% of their scans, all present distolingually, but contradictory results were seen with regards to single-rooted teeth which was seen in 37.9% of the third molars [15]. Radix entomolaris is the term for the presence of an additional root situated distolingually, and this morphological variation has been discovered as a Mongolian characteristic. It is generally an uncommon occurrence for an extra root that is situated in the lingual region of the mandibular third molar [16]. Literature suggests that the overall incidence of the number of roots varied greatly with age. The younger the age, the lower the incidence rate of multi-rooted teeth. No effort was made in the present study to understand this trend. 

When compared to the root canal configuration in the double-rooted teeth, the predominant canal type in the present study on the mesial root was Type II in 67.0% while it was Type I in 79.2% of the distal roots. This finding is similar to the study of Somasundaram reported on 155 scans [8].

C-shaped canal arrangement results from the non-fusion of Hertwig's epithelial sheath [17]. A lingual groove will form if Hertwig's epithelial sheath fails to fuse on the lingual side, and a buccal groove will form if it fails to fuse on the buccal side. As a result, the fusion of the two roots is not uniform, and they are connected by a thin inter-radicular ribbon. These root canals combine to form a wide, slot-like, single, continuous root canal structure that resembles a capital C. The current study reported a total of 21 C-shaped canals in 277 CBCT images evaluated, while the study of Madani identified it in 28 mandibular molars of 154 scans assessed [18].

Though CBCT images are a promising diagnostic imaging modality, certain factors need to be bear in mind for in vivo purposes. In order to obtain higher resolution, effective radiation doses are generally higher with larger FOV, higher Kv, and lower mA thus making the patient prone to greater radiation exposure. In recent times, CBCT machines with lesser exposure mAs are used which in turn reduces dose and risk.

When using a radiographic technique, radiation exposure and the cost incurred to the patient cannot be overlooked. Also, the cost of a CBCT is much higher than periapical radiographs or panoramic X-rays. 

The limitations of the study are that it has a limited sample size and the population is taken from a small area.

## Conclusions

Greater anatomic diversity was seen in mandibular third molars in relation to the number of roots and canal type. When it comes to the third molar teeth that are submitted for the RCT, this is actually a very significant factor to consider because it is based on the particular population. Because of the therapeutic consequences and the significance to anthropology that these variants have, it is essential to have a good understanding of them. The results of the current study can be applied to improve the accuracy of diagnosis and the planning of suitable therapy.
